# Assessing Awareness and Palliative Care Needs in Rural Haryana, North India: A Community-Based Study

**DOI:** 10.7759/cureus.47052

**Published:** 2023-10-15

**Authors:** Ankit Chandra, Rakesh Kumar, Sushma Bhatnagar, Baridalyne Nongkynrih

**Affiliations:** 1 Centre for Community Medicine, All India Institute of Medical Sciences, New Delhi, New Delhi, IND; 2 Onco-Anaesthesia and Palliative Medicine, Dr. B. R. Ambedkar Institute Rotary Cancer Hospital (IRCH) All India Institute of Medical Sciences, New Delhi, IND

**Keywords:** end-of-life care, prevalence, palliation, estimation, supportive care

## Abstract

Background

Community-based palliative care offers a solution to ensure continuity of treatment for patients with life illnesses or life-threatening conditions. Hence, to advocate for community-based palliative care services, it is imperative to generate evidence for palliative care needs in the community. This study aimed to assess the need for and awareness of palliative care in rural Haryana, North India.

Methods

This was a community-based cross-sectional study conducted in six villages of Haryana. A trained investigator conducted house-to-house visits to screen the population for those who require palliative care. The screening was done using three questions, i.e., (1) the presence of a bedridden patient, (2) a person in need of help in activities of daily living, or (3) not able to go to work due to any physical chronic illness. If the answer to any of these questions was positive, a list of patients was prepared, and a community physician trained in palliative care contacted them. Patients and their caregivers were evaluated for their awareness of palliative care through the following question: 'Have you heard about the term palliative care/end-of-life care/home care for bedridden people/community-based care/supportive care?'. Data collection was done using Epicollect5 mobile application, and a descriptive analysis was conducted using the R software.

Results

A total of 1,983 households were visited, and 152 households were excluded as they were found locked during two separate visits. Therefore, a total of 1,831 households, comprising 9,727 individuals, were screened. The need for palliative care was found to be 3.7 per 1,000 population (95% CI: 2.5 - 4.9) and 18.6 per 1000 households (12.4 - 24.8). The most common diseases requiring palliative care were stroke with a focal neurological deficit (27.8%) and cancer (22.2%). One patient with cancer was receiving palliative care. None of the patients or their caregivers was aware of the terms palliative care/end-of-life care/home care for bedridden people/community-based care/supportive care.

Conclusion

The need for palliative care in rural Haryana (North India) was found to be 3.7 per 1000 population. Neither the patients nor their caregivers had an awareness of palliative care.

## Introduction

According to the World Health Organisation (WHO), palliative care is an approach that aims to improve the quality of life for patients and their families who are facing problems associated with life-threatening illnesses by identifying and relieving the suffering by means of early identification and treatment of pain and other problems, whether physical, psychosocial, or spiritual [1]. Palliative care can be integrated with curative treatment for chronic or non-curable illnesses such as terminal cancers, chronic respiratory diseases, heart failure, and others. It can begin from the time of diagnosis until the time of death and bereavement [1,2]. However, the WHO’s definition of palliative care has limitations, which have been addressed by the Lancet Commission report, which expands palliative care as “an essential component of comprehensive care for persons with complex chronic or acute, life-threatening, or life-limiting health conditions that should be practised by all health-care and social care providers and by palliative care specialists, and that can be provided in any health-care setting, including patients’ own homes” [3]. Palliative care is a core component of universal health coverage and yet is often overlooked in practice [4]. To achieve target 3.8 of the Sustainable Development Goals, a country needs to provide palliative care and pain relief [5]. Patients who suffer from life-threatening, or life-limiting health conditions frequently do not receive proper continuity of care. In particular, this is the case in low- and middle-income countries (LMICs), where health services are often overwhelmed by the high demand for care. Community-based palliative care is a way to provide continuity of treatment for patients with terminal diseases. To provide quality palliative care services, it is critical to estimate the need for palliative care.

Globally, it is estimated that 56.8 million people require palliative care annually, with the majority of adults in need of palliative care and children living in LMICs [6]. However, palliative care is available to only 14% of the global population and is predominantly concentrated in European countries [6]. These global estimates are based on modelling done on the data of the global burden of diseases, as there is no primary data collection done for the need estimation of palliative care [6]. In India, a few studies have estimated the need for palliative care and are mostly limited to the southern part of the country [7-9]. These studies in India have used a three-question-based screening tool in the community setting. Using the same tool, we attempted to generate evidence related to the need for and awareness of palliative care in rural areas of Haryana as there is a dearth of evidence from the rural setting of North India. This evidence can provide a clearer understanding of the need for palliative care in India and assist decision-makers in improving palliative care services.

## Materials and methods

**Figure 1:**
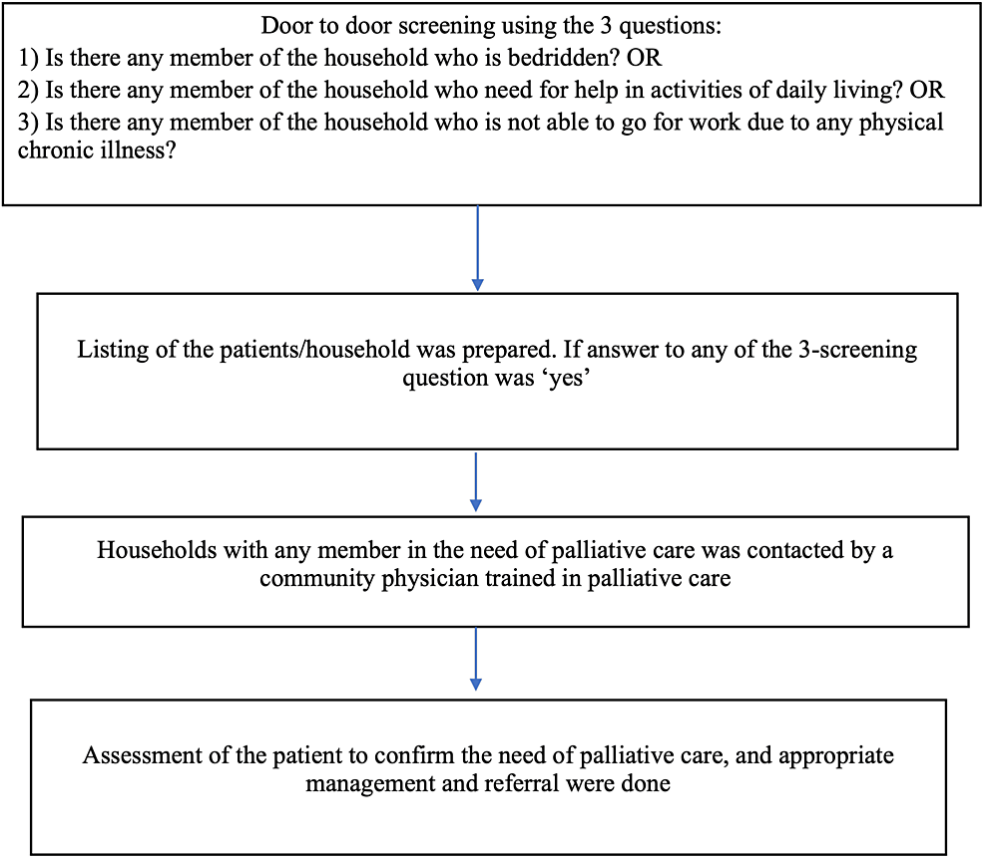
Flowchart illustrating the methodology used in this study to assess the need for palliative care in rural Haryana

A community-based cross-sectional study was conducted in the intensive field practice area (IFPA) situated at Ballabgarh block, Faridabad district, Haryana. The IFPA consists of 28 villages which cover a population of more than 100,000 people. There are around 15,000 households in this catchment area. The area is served by two Primary Health Centres with six subcentres each. This study was carried out from 10th October 2022 to 28th January 2023. Out of the 28 villages, six villages were randomly selected. All households in the selected villages were screened consecutively by a trained data collector who was a local native and received three days of training in palliative care screening. Door-to-door house visits were conducted, and an adult (age ≥ 18 years) informant within the household was selected (giving preference to the head of the household) who could provide information regarding other household members. We excluded houses found to be locked on two separate visits. The screening for the need for palliative care was done by using three questions, i.e., (1) the presence of a bedridden patient, (2) a person in need of help in activities of daily living, or (3) not able to go to work due to any physical chronic illness [7,8,10]. If the answer to any of the three screening questions was 'yes’ for any member in a household, then a line listing of such households was prepared. A community physician trained in palliative care visited the line-listed households to confirm the need for palliative care (Figure 1). To ensure quality control, 5% of all screened houses were cross-verified by the community physician.

Patients identified with palliative care needs were administered a semi-structured questionnaire containing the socio-demographic details, Barthel index for dependency, and disease details. Health records were reviewed to confirm the diagnosis and assess the patient's status. The community physician trained in palliative care provided medical intervention, and appropriate referrals were made when necessary. Patient and their caregivers were also assessed for awareness regarding palliative care by asking “Have you heard about the term palliative care/end of life care/home care for bedridden people/community-based care/supportive care?”. Data were collected in Epicollect5 [11] and analysis was done using software R. Descriptive analysis was done for the socio-demographic and quantitative variables. The prevalence of the need for palliative care was reported with a 95% confidence interval. The problems faced by the caregivers were organized into meaningful themes.

## Results

**Figure 2:**
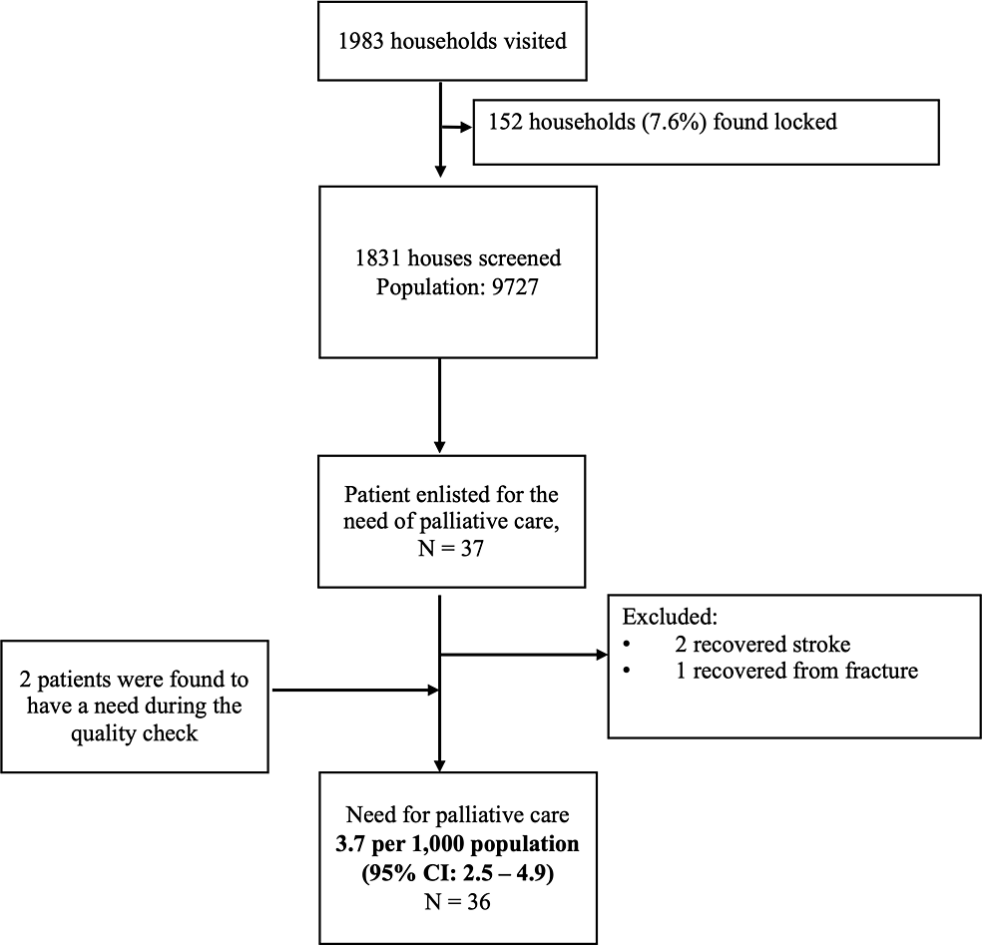
Flowchart depicting the screening process to assess the need for palliative care in the rural population of Haryana.

In total, 1,983 households were visited, out of which 152 households (7.6%) were excluded due to being locked despite two visits (Figure 2). The remaining 1,831 households were screened, which had a population of 9,727 with a mean age of 28.9 years (SD- 19.4) and a mean number of persons per household of 5.3 (SD-1.9). Among the screened households, the majority of the population were males (52.6%), in the age group of 21-30 years (20.1%), and belonged to the upper-middle class (61.8%) (Table 1). Out of the screened households, 36 patients were found to have a need for palliative care, distributed across 34 households. The prevalence of the need for palliative care was 3.7 per 1,000 population (95% CI: 2.5 - 4.9, n=36) and 18.6 per 1,000 households (12.4 - 24.8, n=34).

**Table 1:** Characteristics of the population screened for palliative care need in rural Haryana (N= 9727)

Variable		N (%)
Sex	Males	5,118 (52.6)
	Females	4,609 (47.4)
Age group (in years)	*≤ *10	1,887 (19.4)
	11-20	1,925 (19.8)
	21-30	1,951 (20.1)
	31-40	1,453 (14.9)
	41-50	1,067 (11.0)
	51-60	709 (7.3)
	61-70	488 (5.0)
	>70	247 (2.5)
Socio-economic class	Upper class	164 (9)
	Upper middle class	1,133 (61.8)
	Middle class	362 (19.8)
	Lower middle class	161 (8.8)
	Lower class	11 (0.6)

The study found that patients who needed palliative care had a mean age of 52.3 years (SD - 20.5) and the majority were males (55.6%). The most common conditions that required the need for palliative care were stroke with focal neurological deficit (27.8%) and cancer (22.2%) (Table 2). Of the eight patients with cancer, two had throat cancer, three had breast cancer, one had cervical cancer, one had oral cancer, and one had bone cancer. A total of 11 (30.6%) patients were bedridden, and among them, one patient (9.1%) had bedsores. One patient with advanced-stage throat cancer was undergoing palliative care, primarily pain management using morphine. None of the patients or caregivers was aware of the terms palliative care/end-of-life care/home care for bedridden people/community-based care/supportive care. About 80.6% of patients had a caregiver, and a majority of the caregivers found providing care confining (41.4%) due to the dependency of the patient on daily needs and financial strain (24.1%).

**Table 2:** Characteristics of patients identified with a need for palliative care (n=36) in rural Haryana

Variable		N (%)
Sex	Female	16 (44.4%)
	Male	20 (55.6%)
Current treatment provider	Not under treatment	14 (38.8%)
	Government health facility	9 (25.0%)
	Traditional treatment	7 (19.4%)
	Private health facility	6 (16.8%)
Bedridden	Yes	11 (30.6%)
	No	25 (69.4%)
Presence of bedsore	Yes	1 (2.8%)
	No	35 (97.2%)
Presence of caregiver	Yes	29 (80.6%)
	No	7 (19.4%)
Disease requiring palliative care	Stroke with focal neurological deficit	10 (27.8%)
	Cancer	8 (22.2%)
	Childhood disability / congenital anomaly	4 (11.1%)
	HIV positive	4 (11.1%)
	Neurological deficit (following trauma / slipped disc)	3 (8.5)
	Pelvic Fracture	2 (5.6%)
	Old age weakness with marked dependence	2 (5.6%)
	Chronic kidney disease	1 (2.7%)
	Intellectual disability	1 (2.7%)
	Severe chronic obstructive pulmonary disease	1 (2.7%)

## Discussion

The study found that the prevalence of the need for palliative care in rural Haryana was 3.7 per 1000 population, which is similar to the studies done in rural areas of Tamil Nadu (4.5/1000 population [8], 3.3/1000 population [9]). This prevalence was relatively higher compared to the other studies done in urban areas of North India (2/1000 population in Chandigarh [10], 1.5/1000 population in New Delhi [12]). A study done in the urban part of South India reported the prevalence of the need for palliative care to be 6.1 per 1000 population [7]. The estimation of the need among the four studies is comparable as they all used the same method for screening the community for the need for palliative care [7,8,10,12]. The difference in the findings of these studies can be attributed to the study population and setting (urban or rural). Overall, the prevalence of the need for palliative in the rural setting was much higher compared to the urban setting. Public health programs and policies should consider the unique needs of rural populations and allocate resources accordingly. Most of the patients requiring palliative care had a stroke with neurological deficit similar to other studies [7,8]. We found that the awareness regarding palliative care among the patients and caregivers was nil which was similar to the other studies [9,10,12]. This study highlights the disparities in palliative care needs and awareness between urban and rural areas, emphasizing the importance of tailored public health interventions to improve access to and knowledge of palliative care services.

It was found that one patient with cancer was on oral morphine for pain management and had no therapeutic treatment due to the advanced stage of cancer. However, it was noted that neither the patient nor the caregiver was informed about palliative care/end-of-life care/home care for bedridden people/community-based care/supportive care or any similar terms for the patient's treatment. This shows the deficiency in the health system in informing patients and caregivers about palliative care management. This highlights the reason for low awareness about palliative care in the community. Despite having a National Programme for Palliative Care (NPPC), the efforts to generate awareness regarding palliative care are insufficient [13]. In this modern time, social media can be leveraged for generating awareness [14,15] or the tested method of health awareness campaigns can be stressed [9].

At a global level, various modelling and estimation techniques are employed to estimate the need for palliative care. In 2014, the global need was estimated using the mortality data of WHO Global Health Estimates (2011) which was adjusted by the pain prevalence for 18 diseases, and this number was doubled. This estimated the number of people requiring palliative care at the end of life as 40 million per year [2]. In 2020, the global need was estimated for the second time, based on the serious health-related suffering by the Lancet Commission using the mortality and prevalence data from the Global Burden of Disease Study for 20 diseases plus injuries, and days of suffering for each of 16 identified symptoms. The number of people requiring palliative care (prior to and near the end of life) was estimated to be 56.8 million per year [6]. Considering the world population as 8 billion [16], and the estimated number of people requiring palliative care to be 56.8 million in a year [6], the global need for palliative care can be estimated as 7.1 per 1000 population. However, this figure will vary from year to year. Most of the studies tend to estimate the need for palliative care based on secondary data on mortality or disease prevalence [17-19]. Currently, there is a need for a country-level survey to be done for the estimation of the need for palliative care. There are several validated tools/scales available for screening a diseased/hospitalised patient for the need of palliative care [20-22], but no validated tool is available for screening in a community setting in India. Defining the palliative need solely based on a disease/condition may not be accurate as a person with a life-limiting disease may not have suffering. To obtain a true estimation of the need for palliative care at the population level, there is a need to develop a validated scale/tool to capture serious health-related suffering in the domains of physical, mental, social, spiritual, and caregiver need and use it in national surveys [3].

We conducted a community-based study to estimate the need for palliative care in a rural setting. We experienced a high level of community participation, likely due to the fact that the data collector was a native of one of the selected villages. However, due to limited resources, we were only able to conduct the study in a few villages and interview one individual per household, which may have impacted the accuracy of our findings. Another limitation was locating households for revisits was a challenge that we addressed by utilizing geotagging and contacting a member of the household or their neighbour via mobile phone.

## Conclusions

The need for palliative care in the rural area of Haryana was found to be 3.7 per 1,000 population. The most prevalent conditions requiring palliative care were stroke with focal neurological deficits and various types of cancers. A concerning finding was the lack of awareness of palliative care in the community, highlighting a significant gap in care planning. This underscores the need for increased education and resources to improve palliative care provision in the region.
